# Titin-mediated thick filament activation, through a mechanosensing mechanism, introduces sarcomere-length dependencies in mathematical models of rat trabecula and whole ventricle

**DOI:** 10.1038/s41598-017-05999-2

**Published:** 2017-07-17

**Authors:** Lorenzo Marcucci, Takumi Washio, Toshio Yanagida

**Affiliations:** 1 Department of Biomedical Sciences, Padova University, Via Marzolo 3, 35131 Padova, Italy; 2 Quantitative Biology Center, RIKEN, 6-2-3 Furuedai, Suita Osaka, 565-0874 Japan; 3 Graduate School of Frontier Sciences, The University of Tokyo, 5-1-5 Kashiwanoha, Kashiwa-shi Chiba-ken, 277-8561 Japan

## Abstract

Recent experimental evidence in skeletal muscle demonstrated the existence of a thick-filament mechanosensing mechanism, acting as a second regulatory system for muscle contraction, in addition to calcium-mediated thin filament regulation. These two systems cooperate to generate force, but the extent to which their interaction is relevant in physiologically contracting muscle was not yet assessed experimentally. Therefore, we included both regulatory mechanisms in a mathematical model of rat trabecula and whole ventricle. No additional regulatory mechanisms were considered in our model. Our simulations suggested that mechanosensing regulation is not limited to the initial phases of contraction but, instead, is crucial during physiological contraction. An important consequence of this finding is that titin mediated thick filament activation can account for several sarcomere length dependencies observed in contracting muscle. Under the hypothesis that a similar mechanism is acting on cardiac muscle, and within the limits of a finite element left ventricle model, we predict that these two regulatory mechanisms are crucial for the molecular basis of the Frank-Starling law of the heart.

## Introduction

Muscle force is directly proportional to the number of myosin motors going through the cross-bridge cycle, the ATP-driven interaction between myosin proteins protruding from the thick filament and actin proteins forming the thin filament. The cross-bridge cycle involves the following: formation of the actomyosin complex through attachments between the two proteins; one or more force-generating changes of configuration in the actomyosin complex, using ATP as energy; and disruption of the complex by detachment between the two proteins, a process favored by binding of a new ATP molecule to the myosin motor. The number of force-generating cross-bridges is known to be regulated by calcium signalling. The action potential triggers release of calcium ions into the myofibrillar space, and these can then occupy troponin calcium-binding sites. This shifts the tropomyosin filaments, uncovering the myosin-binding sites on the thin filaments and enabling cross-bridge formation. In this process, at higher calcium concentrations [Ca^2+^] in the myofibrillar space, there is a higher probability for available myosin motors to produce force.

**Figure 1:**
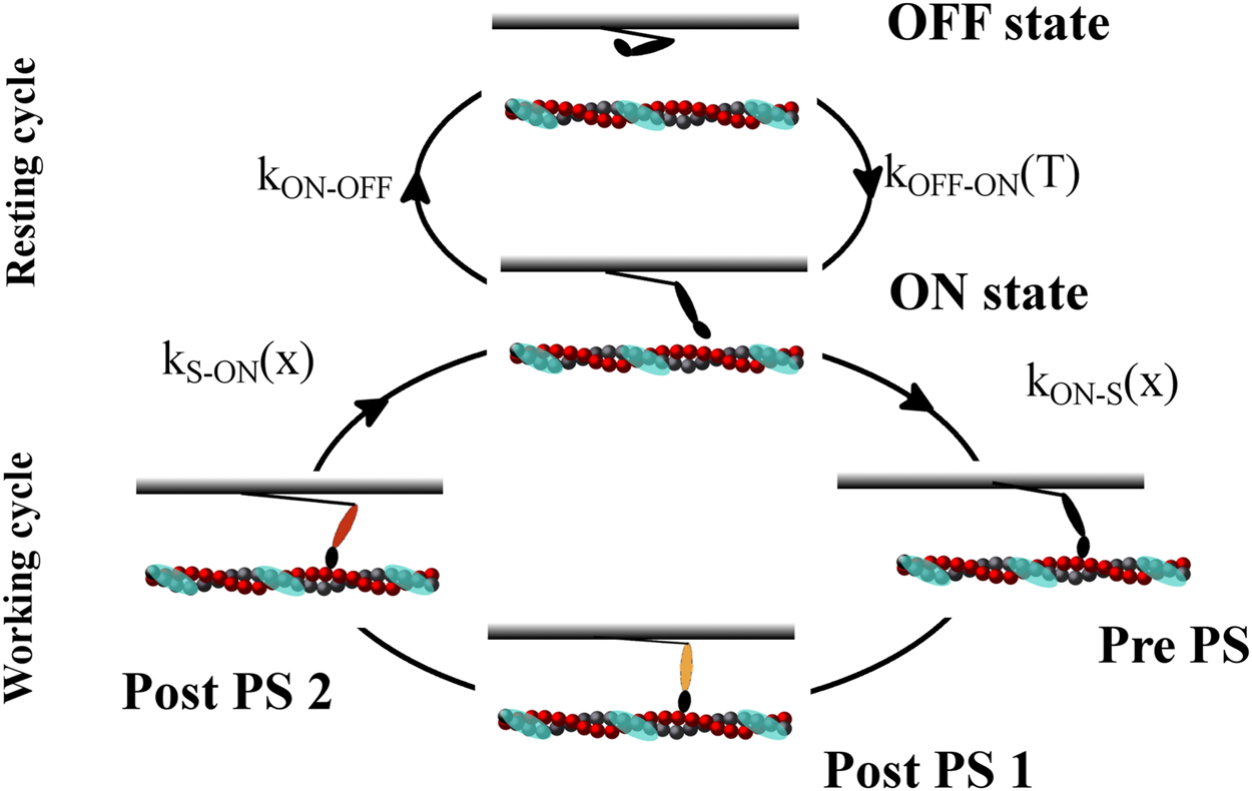
Two-cycle model. In the model, myosin motors can follow two cycles, a non-force-generating, “resting” cycle and a force-generating, “working” cycle, connected through one motor state. In the resting cycle, motors can lie along the thick filament in the OFF or super-relaxed state, or they can rise toward the thin filament, in the ON or active state, ready to enter into the working cycle. Motors in the ON state can use energy from ATP to generate active force. Myosin motors can then strongly attach to actin filament (shown with the periodic troponin-tropomyosin units in cyan) and perform a two-step power stroke. The rate constant from the OFF to ON state, k_OFF–ON_, is a function of the tension, T, acting on the thick filaments, indicating a mechanosensing mechanism, while k_ON–OFF_ is a constant. Rate constants for strong attachment (k_ON–S_) and detachment (k_S–ON_) are functions of the axial position of each motor, with respect its relaxed state, as in the original Huxley 1957 model^35^.

Recent experimental evidence in skeletal muscle showed the presence of a second regulatory mechanism in muscle contraction, in addition to calcium-mediated signalling for thin filament activation. The second mechanism regulates the amount of motors ready to work, through thick filament activation. In fact, cyoEM and X-ray analyses substantiated the existence of two resting states of thick filaments. Detached myosin motors can lie along the thick filament^1–3^ in a state consuming low ATP. To generate force, these “super-relaxed” or “OFF” myosin motors must be activated, switching into an active or “ON”^4, 5^ state. In this state, motors are in a more disordered configuration and have a higher ATP consumption rate. Linari and co-workers used X-ray diffraction and mechanical methods on tetanized, intact frog skeletal fibers. They showed that, at higher active tensions sustained by the thick filaments, there was a higher probability that OFF myosin motors would be recruited to the ON state. This proved the existence of a mechanosensing (MS) mechanism acting on the thick filament^4^. Recently, using probes attached to myosin motors in skinned rabbit skeletal fibers, Fusi and co-workers shown that the same effect was generated by passive tension transmitted to thick filaments through the giant visco-elastic protein titin^5^, which runs from the Z-lines of the sarcomere to the M-lines along the thick filament. That study, using solutions with activating levels of Ca^2+^ and blebbistatin, indicated that, as a regulatory system, the MS mechanism acts independently of calcium signalling. In the emerging picture, the conventional cross-bridge cycle is then separated into two parts: a non-force generating, or “resting”, cycle, including the OFF and ON states, and a force generating, or “working” cycle, including the ON and strongly-attached states (Fig. 1). Force regulation in contracting muscle includes both cycles, through thick filament activation, mediated by passive and active tension, which recruits active motors, and thin filament activation, mediated by [Ca^2+^], which regulates how many active motors can go through the cross-bridge cycle to generate force.

This “two-step” regulatory system may be partially hidden in common skeletal muscle analyses, which are usually activated at a constant [Ca^2+^], often in tetanus, and at a sarcomere length (SL) where the passive tension is negligible, near the plateau region of the force–length relationship. On the contrary, in cardiac muscle contraction, there are rapid variations in [Ca^2+^] and relatively high passive forces at physiological SLs^6–8^, because of the shorter length and higher stiffness of cardiac titin. For this reason, we analysed cardiac muscle, extrapolating the MS properties from behaviours observed in skeletal muscle. Experimental evidence for the existence of the MS mechanism in rat cardiac muscle was recently obtained by Reconditi and co-workers^9^. In our study, we estimated the importance of the cooperative regulation of thin and thick filaments, in physiological situations, using mathematical models of rat ventricular trabecula and whole ventricle. We included both, and no additional, regulatory systems in the model. We used parameter values that were the same orders of magnitude as the experimental data and accounted for the titin passive tension in thick filament activation. The simulations predicted that cooperation between the two regulatory systems is not limited to the early phases of contraction, but that thick filament activation also contributes to regulating the number of active motors during physiological contraction. The most important consequence was that the simulated effect of titin mediated thick filament activation plays a fundamental role in contracting muscle. Our findings can explain the SL dependence on active force generated in isometric and isotonic contractions. Moreover, as an emerging property of the cooperativity, the interaction of the two mechanisms explains the SL dependence of myofilament calcium affinity, without requiring *ad-hoc* hypotheses of [Ca^2+^] dependence. Notably, SL effects on both force generation and myofilament calcium sensitivity were proposed to contribute to the Frank-Starling law of the heart. We then included our trabecula model in a finite element (FE) rotationally symmetric model of the rat left ventricle, showing that titin mediated MS activation can, at least qualitatively, account for the Frank-Starling curve.

## Model and Results

### Model description

New experimental evidence changed the conventional description of the cross-bridge working mechanism. We correspondingly adapted our model (Fig. 1 and Methods) and report here its major differences from more conventional models. To reproduce the OFF state and the MS mechanism, myosin motors move along two different cycles, a “resting” cycle between the OFF and ON states, and a more conventional “working” cycle, which shares the ON state with the previous cycle. Transition kinetics in the resting cycle is characterised by a tension dependent forward rate (MS mechanism), to be described further in this report, and a constant backward rate. In the working cycle, myosin motors generates force through attachment and detachment from the thin filaments. Working cycle parameters are defined to fit the experimental data reported in rat trabeculae^10^ (see next section). Filament lengths and sarcomere geometries are crucial aspects of the model, because they may also account for the SL dependence of the force generated. Despite that, in cardiac muscle, effects of these parameters appear to be limited, because of its shorter thin filament lengths^8^. With the imposed values, the potential availability of myosin motors was constant for SL between 1.8 and 2.2 µm.

For the following analysis, we modified a model recently proposed by one of the authors, the first to include the MS mechanism in conventional muscle models^11^. Compared with conventional models, the main novelties were: (i) from the attached state, myosin motors move to the ON state, where they can switch OFF or start a new working cycle; and (ii) the rate of switching ON from the OFF state (k_OFF-ON_) depends on the tension acting on the thick filament, both active and passive. The previous work described effects of the MS mechanism on kinetics of force development in frog skeletal muscles, when exposed to a high and constant (tetanizing) calcium concentration, and at SLs, where the titin passive force should be negligible (2.14 μm). In the current study, we also introduced the effects of titin, summing a variable passive tension at different SLs with the active tension generated by the attached myosin motors. While a simpler (linear) tension dependence of myosin activation was introduced, as in the new experimental data from Fusi and colleagues on skeletal muscle^5^, titin passive tension was imposed, following experimental data from rat cardiac muscle. Moreover, experiments in skeletal muscles were made with single fibers, though myofilaments occupy only about one half the cardiac trabeculae cross-sectional area. Then, the tension simulated by the sarcomere model was divided by 2. Compared with the original model, a more detailed fitting of thin filament regulation, through activation of the troponin-tropomyosin (TT) units, was also required to reproduce the twitches during the imposed Ca-transients. Finally, myosin motor kinetics were modified to account for the difference in muscle type and temperature in our experimental system, compared with in previous systems.

Following experimental observations in rabbit skeletal muscle^5^, k_OFF-ON_ is defined as a piecewise linear function of the tension acting on each thick filament. Lack of experimental data on the MS mechanism in cardiac muscle is a limitation of our study. We based our analysis on the available data, avoiding further hypotheses. Different values may also be consistent with the experimental data, but we limited our analysis to the maintaining the same order of magnitude, because of differences in muscle type and temperature. Tension generated by titin is assumed to be zero for SL values equal to or lower than 1.9 µm, and increases with the square of SL to generate 9 kPa at SL = 2.2 µm, similar to experimental data in mouse cardiac myocytes^7^.

Thin filament activation is regulated by the [Ca^2+^] by the presence of TT units. When a TT-unit is not activated, the probability of strong attachment in the corresponding thin filament region is decreased by a constant value, μ_Ca_^12^.

### Titin-mediated mechanosensing activation plays a role in single twitches

**Figure 2:**
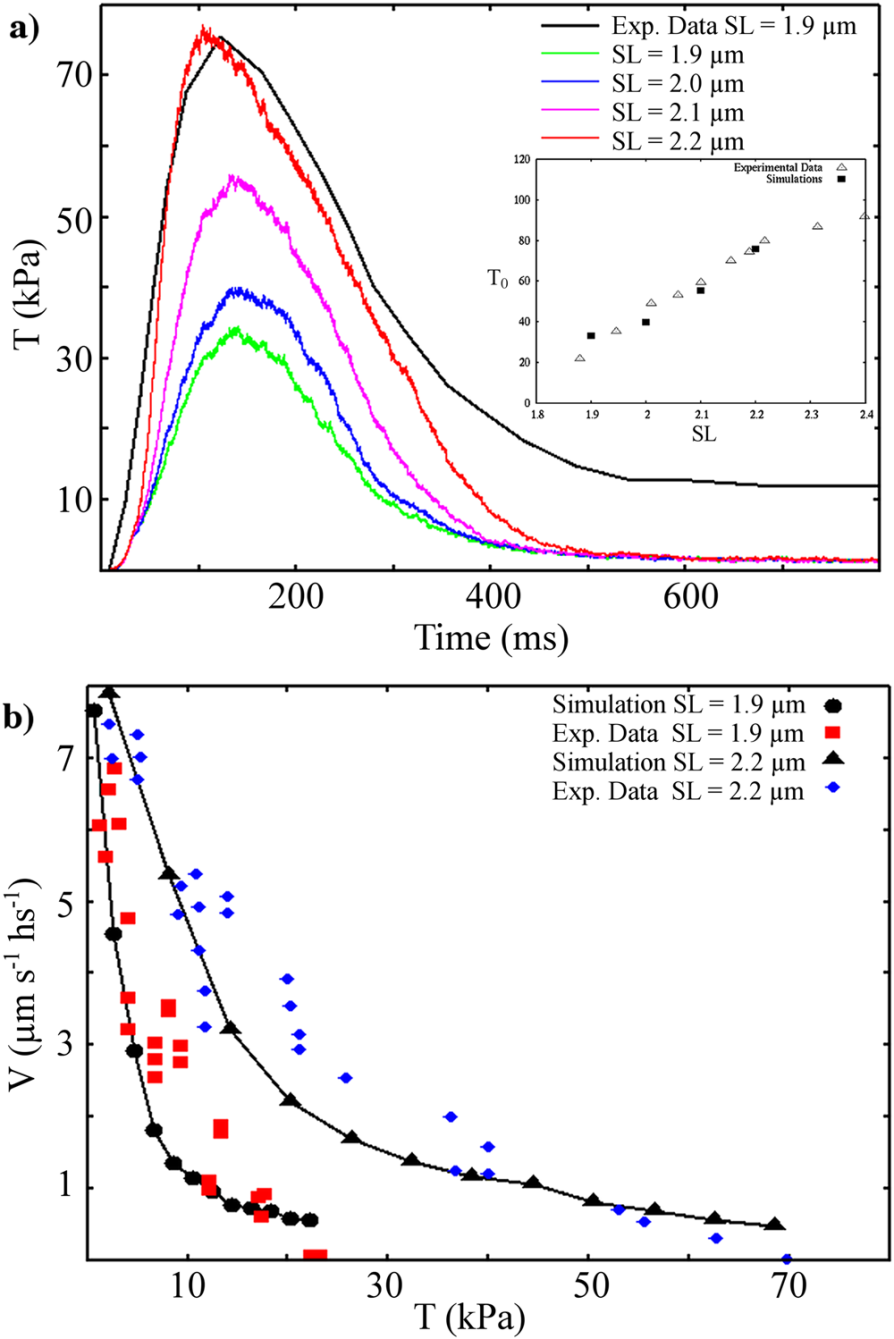
Simulations of isometric and isotonic twitches. Upper: Simulation of the tension generated during a physiological [Ca^2+^] transient in isometric conditions, at different SLs. The experimental time transient at SL = 2.2 µm (black line) is scaled to the simulated maximum tension at the same SL (red line). Simulated maximum active tensions *vs*. SLs are shown in the inset and compared with experimental data (simulations, filled squares; experimental data, empty triangles). Lower: A steady velocity of contraction is reached under isotonic conditions imposed near the maximum tension of a twitch. The simulated force–velocity curves (black dots, SL = 1.9 µm; black triangles, SL = 2.2 µm; and lines) quantitatively fit the experimental data, at two different SLs, without modifying the mechanokinetics properties of the motors. All experimental data are from Caremani *et al*.^10^.

We first used the model to simulate published data obtained in electrically stimulated intact rat trabeculae^10^. In that work, the mechanokinetic properties of myosin were analysed *in situ* using a striation follower, allowing an unusual level of precision in SL regulation. This was crucial for our study. We set our parameters in the working cycle to reproduce the maximum force in twitches at SL = 2.2 μm, using calcium transients, as obtained from the cell model proposed by Tusscher and co-workers^13^ (see Supplementary Information, and Supplementary Fig. S1, inset). Then, we simulated force during isometric contractions at different SLs, which means at different passive tensions (Fig. 2a). The model succeeded in quantitatively predicting increasing maximum tension during twitches, at increasing SLs (see inset in Fig. 2a, only active tension is reported in the figure, consistent with the experiment). At SL = 1.9 μm, without any passive tension, thick filament regulation generates about 30% of active (ON) myosin, from which thin-filament regulation recruits about 14% of force-generating myosin at the tension peak (see Supplementary Fig. S2). At SL = 2.2 µm recruitment of active myosin more than doubles (66%), as does that of force-generating myosin (33%). Notably, this implies a constant duty ratio at different SLs, when corrected by the MS mechanism as an active/attached myosin ratio, instead of the usual total/attached myosin ratio. This is in strong agreement with the experimental results of Reconditi and colleagues^9^. Moreover, the simulated ratios reported here refer to myosin dimers (see Methods). When corrected to myosin motors, the ratios are very close to those inferred from a structural model applied to X-ray diffraction data (Model 2 and Table 1, in Reconditi *et al*.), which reports 8 and 18% of strongly attached motors under fixed end and length clamp conditions, respectively. These protocols are approximately related to our SL values of 1.9 and 2.2 µm, respectively. The previously reported data corresponded to 16 and 36% of myosin dimers, compared with our values of 14 and 33%. Myosin in the OFF state would imply that both motors are in the OFF state. Therefore, experimental values of 15 and 64% may be compared with our simulated values of 30 and 70% for SL of 2.2 and 1.9 µm, respectively.

The model predicts that, with a passive tension increase of less than 0.1 T_0_, the positive feedback between the two regulatory systems would increase the maximum tension more than two-fold, supporting the importance of this cooperation in actively contracting muscle. This effect is related to the positive feedback between calcium-mediated thin filament regulation and tension-mediated thick filament recruitment of active motors.

We extended our simulations to isotonic contraction, to reproduce the force–velocity curve. With the same set of parameters, the model can reproduce velocities of contraction at different levels of external force for two SLs, still showing similar maximum velocities (Fig. 2b). In agreement with experimental observations^10^, the normalised force-curves are then similar at different SLs, because the various behaviours were obtained without modifying the motor mechanokinetic properties. Instead, the force–SL relationship is determined by a modulation of the number of active myosin motors, generating different total tensions once exposed to the same thin filament activation.

In the model, the SL has an effect only on recruitment of active motors. Therefore, simulating different behaviours at different SLs during twitches, the model shows that thick filament regulation is still functional during active contraction. The positive feedback between the two regulatory systems is, therefore, not limited to the early phases of tension generation and its kinetics^4, 11^.

### SL dependent myofilament calcium sensitivity is property arising from the positive feedback of thin and thick filament regulation

**Figure 3:**
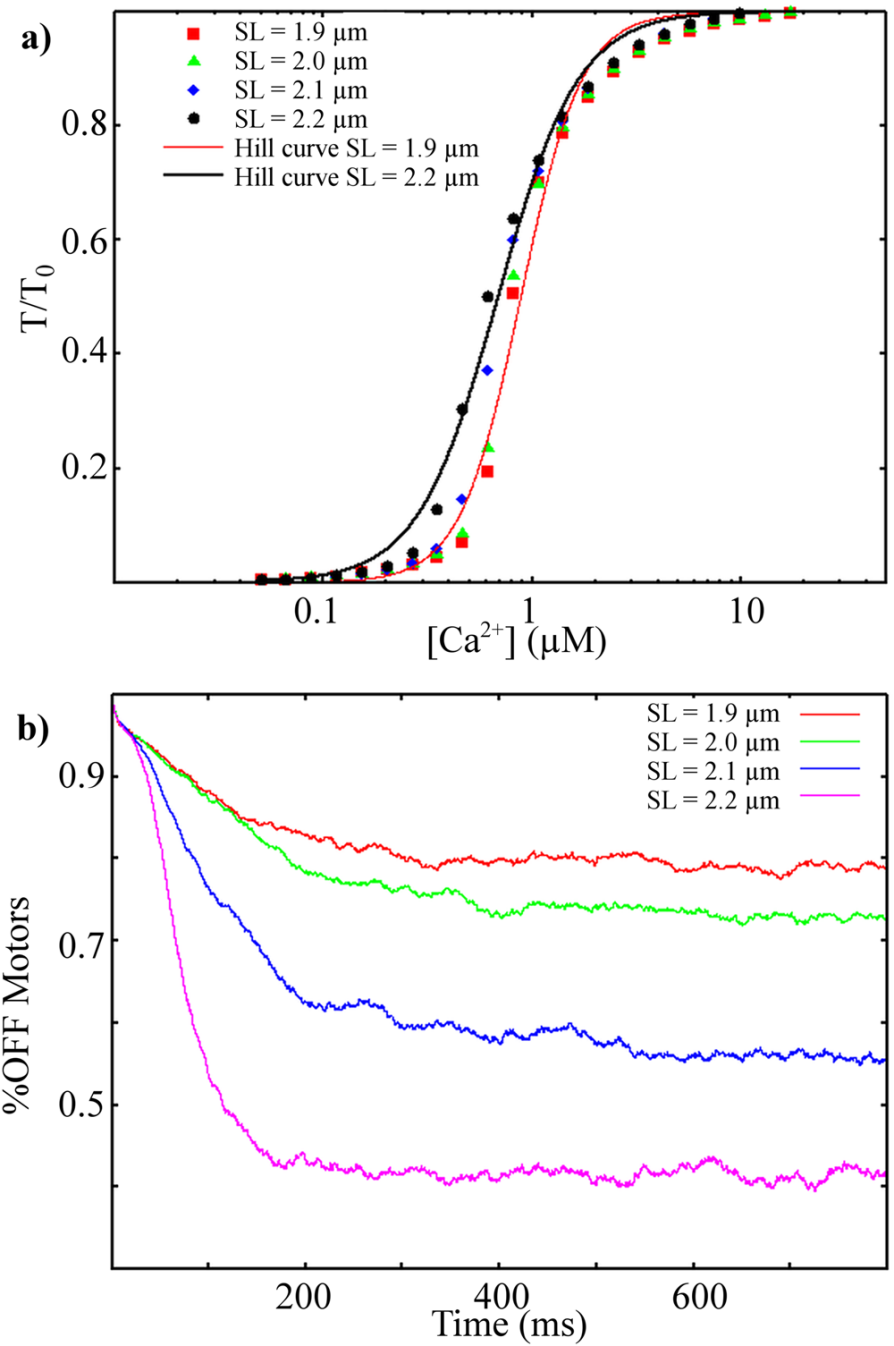
Simulations of force–pCa curves at different SLs. Upper: Maximum force at different [Ca^2+^] values, without titin tension (at SL = 1.9 µm, red dots) and with increasing titin tensions at higher SLs. Force–pCa curves are shifted to the left at higher SLs, as was also observed experimentally. Continuous lines represent the best fits with Hill’s curves, for SL = 1.9 μm (red) and SL = 2.2 μm (black). Bottom: Relative number of motors in the OFF state at steady [Ca^2+^] = 0.6 µM. The higher number of active motors at higher SLs, generating higher steady values of attached motors and, thus, higher active tension values, shifting the pCa–force curve to the left.

We extended our analysis to the force generated at constant [Ca^2+^] values, reproducing the force–pCa curve at SL = 1.9 µm, therefore, without a titin effect (Fig. 3a, red squares). Fitting the data to a Hill’s curve (red continuous line), we obtained nH = 3.0, not far from experimental values^14^.

Experimental data in skeletal muscle, at tetanizing [Ca^2+^], showed that the MS mechanism “saturated” at approximately 0.5 T_0_^4^. At this value, the MS mechanism, induced by active tension, recruits more active motors which, in turn, increases active tension, generating an avalanche of active motor recruitment, up to a maximum. Nevertheless, at lower [Ca^2+^], the model predicts that, even when the two regulatory systems lead to a relatively high active tension, the MS mechanism is still involved in recruitment of active motors.

Next, we tested the cooperative effects of the two regulatory systems, when both active and passive tensions stretch the thick filament, simulating the force–pCa curves at different SLs, from 1.9 to 2.2 µm (passive tensions from zero to 9 kPa, respectively). At low [Ca^2+^] (Fig. 3a), thin filament regulation maintains tension below the threshold for activation of the MS mechanism. At very high [Ca^2+^], the MS mechanism reaches its limit in recruiting active motors (in the model, this is approximately 85% of total motors). At intermediate [Ca^2+^], the higher passive forces at higher SLs, despite being lower than the active force, still increase the number of available active motors which, in turn, generate a higher number of attached cross-bridges. The relative number of motors in the OFF state reaches a lower steady state value at higher SL levels (0.79, 0.73, 0.56 and 0.41 at SL 1.9, 2.0, 2.1 and 2.2 µm respectively, Fig. 3b). Therefore, without including other effects on thin-filament activation, the model predicts that, at higher SLs, the force–pCa curve would shift to the left, resulting in an apparently increase in calcium sensitivity of the myofilaments. This is a property arising from the co-existence of the two regulatory mechanisms. The pCa_50_ shifts from 6.06, at SL = 1.9 µm, to 6.16, at SL = 2.2 µm, with a ΔpCa_50_ = 0.1 pCa units. Even though the parameters were defined to reproduce trabeculae tensions at different velocities during twitches, the model predictions regarding the force–pCa curve were compatible with the experimental data. In mouse skinned cardiac myocytes, a reduction of passive tension, associated with ΔpCa_50_ = 0.15 pCa units, was observed with lengthening from SL = 2.0 µm to SL = 2.3 µm. This reduction was lowered to 0.06 pCa units in the presence of dextran, which leads to a more physiological interfilament spacing^15^.

Several experimental studies showed the effects of the passive tension or titin isoforms on calcium sensivity of the filaments^15–17^. Interestingly, it was hypothesised that the probability of force-generating actomyosin interactions would be increased by increased myosin motor disorder, and, consequently, ATP-ase activity^18^. Our results were also in agreement with observed decreased effects of the SL on shifting of the force–pCa curve in the presence of trypsin, an enzyme that degrades titin^15, 17^.

The limitations of this model are apparent in the simulated force-pCa curves. First, the same tension at high [Ca^2+^] is predicted at different SLs, while, experimentally, the maximally activated tension also increases with SL^18, 19^. Second, the nH parameter in the Hill’s curve decreases at higher SL (nH = 2.2 at SL = 2.2 μm), contrary to what was observed experimentally in cardiac muscle. Moreover, predicted pCa_50_ values are less than those determined experimentally in rat trabeculae^17^ (experimental data not shown). These limitations are described in the Discussion. They were likely caused by our choice to include only two regulatory mechanisms in our model. Despite these limitations, we believe that using the simplest hypotheses, thus avoiding further assumptions, was a useful approach for appreciating the interaction between the two mechanisms.

### Mechanosensing recruitment of active motors can contribute to the molecular basis of the Frank-Starling law of the heart

**Figure 4:**
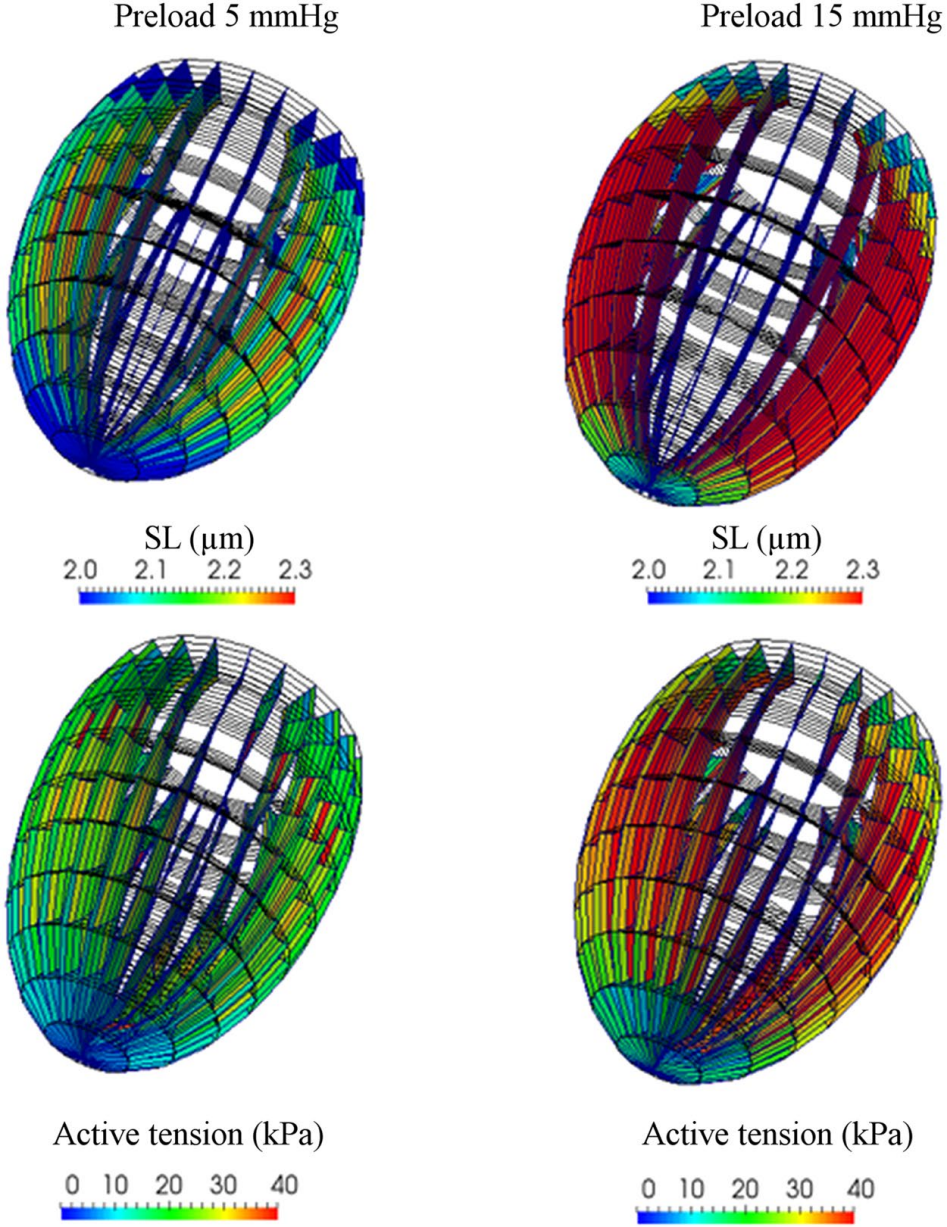
Ventricle model simulations: Sarcomere lengths in different finite elements at the end of the diastolic phase (upper part) and active tension at peak (lower part, at 0.1 s after the beginning of the systolic phase). Two preloads are shown, at 5 and 15 mmHg (left and right, respectively). The tension is higher at 15 mmHg because of the effects of titin on the mechanosensing mechanism.

We showed that the cooperativity between the MS mechanism, mediated by titin passive force, and calcium activation of the thin filaments, can affect SL-dependent force and myofilament calcium sensitivity. These two effects were correlated to increased ejection volume at increased end diastolic volumes in the ventricles, the so-called Frank-Starling law of the heart^15, 17, 20, 21^. We now include our trabecula model in a rotationally symmetric finite-element model (see Methods, Supplementary Information and Fig. 4 and Supplementary Fig. S1) of the rat left ventricle, to predict its behaviour at different preload pressures. Even though many other effects probably cooperate to sustain this important cardiac function, we included only these two regulatory mechanisms to estimate their impacts on total ventricle behaviour.

**Figure 5:**
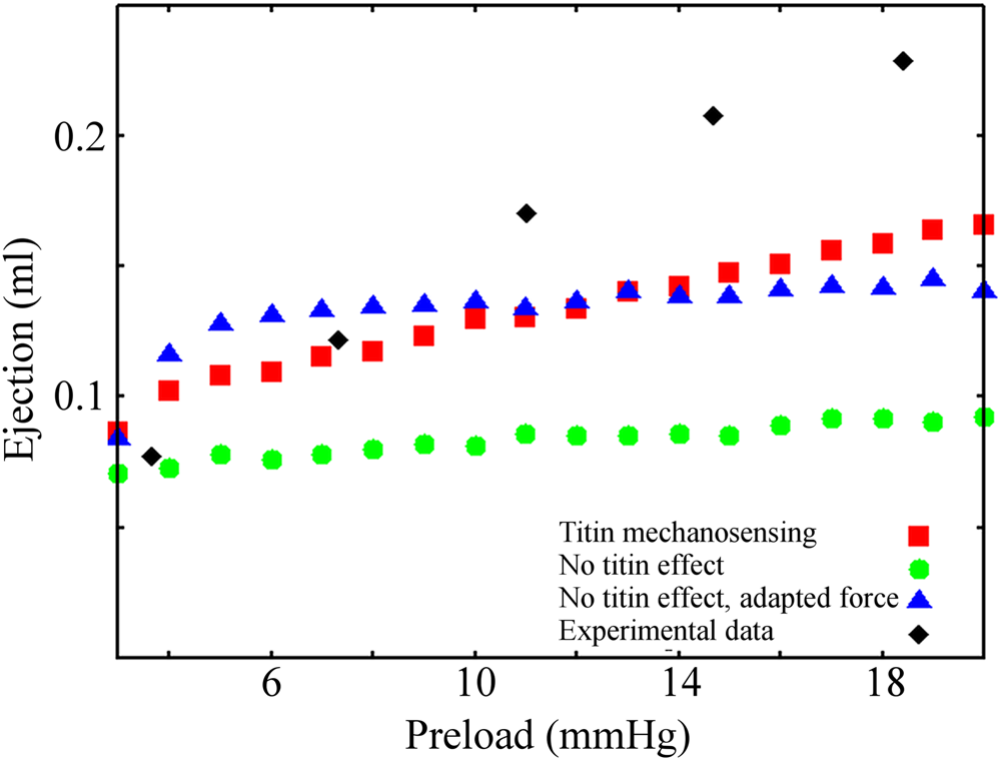
Left ventricle FE model simulations. Ejection predicted by the rotationally symmetric FE model of the rat left ventricle, against increasing preloads with the double-cycle model (red squares), compared with the experimental data (black diamonds). The ejection increases at higher preloads, showing that the model can reproduce, at least qualitatively, the Frank-Starling law. The passive tension effects of titin on the mechanosensing mechanism are eliminated from the model in two ways (blue triangles and green dots). Neither predicts an increased ejection, except for small preloads, which lead to decreased overlap of the two filaments.

The ventricle model, with the titin mediated MS mechanism, reproduces the increased tension observed at increased preloads (Fig. 4). The effect is related to the higher SL, which leads to a greater number of active and force generating motors. Moreover, even at preloads that decrease filament overlapping (Fig. 4, right), the number of active motors still increases because of the MS mechanism, generating higher tension. The Frank-Starling law is then reproduced with the MS model, including titin passive tension (Fig. 5, red squares). We then modified the model, such that passive tension does not affect the activation of myosin motors, both using unmodified attachment and detachment rates (green dots), which has a peak in tension in the MS model with SL = 1.9 µm, and with rates modified to generate a peak in tension (blue triangles), corresponding to SL = 2.1 µm. In both cases, the predicted ejection is constant when the preload leads to a completely overlapping geometry of the sarcomeres. Simulations were also compared with experimental data^22^ (black diamonds), showing a good fit at low preloads. The discrepancies in fit at higher preloads probably were attributable to involvement of other regulatory systems, like thin-filament regulation and lattice-spacing effects, not included in our model. This left ventricle FE model predicts, at least semi-quantitatively, that the MS-related regulatory system would play a crucial role in the molecular basis of the Frank-Starling law of the heart.

## Discussion

Recent experimental evidence improved upon the classical view of the “cross-bridge cycle”. In its classical description^23^, the stable states of the myosin motors follow one cycle where, despite the possibility of back and forward transitions, it is required that motors pass through all steps. However, the emerging concept instead, indicates two interconnected cycles (Fig. 1), sharing the active or ON state. From this state, myosin motors can perform a force-generating, or “working”, cycle, similar to the conventional cross-bridge cycle, with several intermediate stable states, or it can be in a “resting” cycle, switching to the OFF state. The interaction between these two cycles, with increased complexity over that of the one-cycle hypothesis, allows for finer regulation and emerging properties. In this new description, the players are more independent of one another. The working cycle is governed by calcium dependent activation of the thin filaments and the resting cycle by the tension, both active and passive, sustained by the thick filaments. That is, the MS mechanism regulates the available myosin motors, while [Ca^2+^] regulates the number of active motors generating force. The model presented in our study predicts that the two regulatory mechanisms would cooperate, not only during the initial phases of the contraction but also, and mainly, during the active contraction, both during twitches and at constant [Ca^2+^] during submaximal activation. This cooperation, through titin, can explain the apparent increase in myofilament calcium sensitivity and the SL dependence of tension generated during a twitch.

Our model has some limitations in the simulation of the force–pCa curve at different SLs. This is likely because the model does not include several mechanisms for thin filament-mediated regulation of length dependent activation in the heart and the slope of the force-pCa curve^24–28^. These include synergic interactions between myosin, actin and troponin C, as well as cooperative myosin–myosin and myosin–actin effects, for instance through displacement of tropomyosin filaments^29, 30^. Terui and colleagues showed, through a mathematical model, the relationship between length dependent activation in the heart and interfilament lattice spacing modulation^27^. Recently, Zhang and colleagues proved a direct modulation of thin-filament activation by the SL, which was effective even in the absence of force-generating myosin heads^28^. Moreover, titin stiffness varies with [Ca^2+^], affecting the MS mechanism^5^, while, for simplicity, we imposed a titin passive tension based on data from relaxed muscle. Another interesting explanation of the limited force-pCa simulation is related to our hypothesis that, at high [Ca^2+^], the active tension “saturates” the MS mechanism and no differences can be observed at different SLs. This is how the MS mechanism appears to work in skeletal muscle^4^, but this saturation effect may not be present in cardiac muscle where, for physiological reasons, the MS mechanism may be active until maximum tension is generated. To fit the experimental twitches, still neglecting such cooperative mechanisms, we had to modify the sensitivity of actin filaments to [Ca^2+^] in the component not related to SL. This explains the lower pCa_50_ predicted by the model, with respect to experimental data, and why nH is inversely proportional to SL (see SI text). However, the mechanisms of thin and thick filament cooperation are still under debate, requiring different hypotheses in mathematical modelling. Our results, despite the limitations introduced by our relatively simple hypotheses, strongly supported the idea that the mechanosensing mechanism, and the cooperation which it naturally introduces, play crucial roles in the force-pCa shape and should be included in mathematical models intended to analyse this feature.

The adaptability of cardiac contraction force to external conditions occurs through the very complex, multiscale structure of the heart. Modifications at the macroscopic level, change behaviours at the level of single molecules which, in turn, affect the performance of the whole heart during every beat. A multiscale approach is, thus, needed to fully understand the physiology of cardiac contraction. Mathematical models can fill the gaps between experimental data obtained from single molecules and whole hearts. One of the most important adaptive behaviours is the Franck-Starling law of the heart. Heart muscle increases its contractility when an increased venous return dilates the ventricle more, balancing the blood volume ejected during systole with that received during diastole. This is necessary to achieve steady state^31^. This behaviour resembles the well-known force–length relationship in skeletal muscle, generated by various overlapping regions of thin and thick filaments, at different SLs, a process modulating the fraction of myosin available to interact with actin filaments^32^. However, correlating the cardiac sarcomere geometry and filament lengths with the regions of interest in the cardiac SL–tension relationship, it is evident that myofilament overlap cannot fully explain this relationship and that additional factors must be considered^24^. Among others, two important aspects were highlighted as playing key roles: the passive tension generated by titin and myofilament Ca^2+^ sensitivity^15, 17, 20, 21, 33, 34^. Our simulations showed that the MS mechanism can quantitatively link these two aspects and explained the different tensions generated at various SLs in cardiomyocytes, assuming that this process is also relevant in intact cardiac muscle. Thus, these two regulatory processes, linked through titin mediated passive tension, may represent an important aspect of the molecular mechanism of the Frank-Starling law. Recently, the contribution of titin strain to Frank-Starling law was demonstrated experimentally by Ait-Mou and co-workers^21^, using X-ray diffraction and X-ray electron density. SL was believed to affect the distribution of myosin motors among various structural states. In our study, we quantitatively substantiated this hypothesis with an *in-silico* method, relating it to the MS mechanism. Moreover, Ait-Mou *et al*. associated a reduction of I_11_/I_10_ ratio in X-ray diffraction with a higher degree of order in the thick filament at longer SLs in diastole, appearing to discard the hypothesis of closer positioning of myosin motors toward the thin filament at higher SLs. Our model is not in contradiction with this observation. In fact, imposing a minimum level of tension (T_min_ = 17 kPa) to activate the MS, greater than the passive tension at high SL (T_p_ = 9 kPa at SL = 2.2 μm), our model predicts the same amount of myosin motor in the ON state at any SLs in diastole. The increased calcium sensitivity arises from the higher passive tension only when the active tension is increased over T_min_ − T_p_. A minimum tension to activate the MS mechanism was also demonstrated in skeletal muscle^4^. In contrast, Zhang and co-workers showed a length-dependent change in orientation of myosin motors at low pCa^28^ in demembranated ventricular rat trabeculae. Our model cannot yet quantitatively discriminate among these hypotheses and experimental data on the MS mechanism in cardiac muscle are only very recently becoming available. Despite that uncertainty, both *in situ* and *in silico* methods strongly supported the importance of the MS mechanism in the molecular basis of the Frank-Starling law in the heart and iterative feedback between experiments and modelling will add insights in the future. For instance, perturbing the super-relaxed state, without affecting other muscle properties, would allow our conclusions to be tested and, if confirmed, would provide important information for the study and the treatment of related cardiac pathologies. In fact, any pathology related to the fine recruitment of active motors might be hidden in maximally activated sarcomeres, when active tension would be saturating the MS mechanism.

## Material and Methods

### Sarcomere model

Each myosin head follows the two-cycle scheme shown in Fig. 1, with a super-relaxed or OFF state in equilibrium with an active or ON state, and three stable strongly attached states again in equilibrium with the ON state. K_OFF–ON_ has a minimum value, k_min_, up to 17 kPa on the trabecula cross-sectional area (*i.e*., 34 pN per thin filament or 68 pN per thick filament in the model). This tension value is less than 15% of the maximum tension generated by the model, under high calcium conditions at 27 °C (T_0_ = 120 kPa on the trabecula cross-sectional area). Experimental data in skeletal muscles showed a threshold value for activating tension of about 0.1 T_0_^4^. The value of 0.15 T_0_ is also close to the lower values reported by Fusi and co-workers^5^. K_OFF–ON_ reaches its maximum value, k_max_, at 12 kPa, close to the maximum value analysed experimentally^5^. The minimum and maximum rates are k_min_ = 10.2 ﻿s^-1﻿^ and k_max_ = 442 s^−1^, respectively. Numerical values were chosen to accommodate the fitting of tension generated by rat trabeculae at SL = 2.2 µm, but close to values observed in rabbit skeletal muscle^5^, where the rate constants of transients in the analysed order parameter range from 100 to 500 s^−1^, as force is increased from 20 to 300 pN per thick filament. The k_ON–OFF_ rate is assumed to be constant, at 262.4 s^−1^ (the experimental value was 400 s^−1^ and was relatively independent of tension). The attachment and detachment kinetics are governed by the classical hypothesis of the Brownian search and catch mechanism, originally proposed by A.F. Huxley^35^. In the attached state, myosin motors can perform a two-step power stroke, with dynamics governed by a Kramers’ approximation of the diffusion into a multi-stable energy, as defined in our previous model^36^. The dynamics of each myosin motor is followed by a Monte-Carlo method, based on a previously described MS model^11^, with a time step Δt = 1 µs. Myosin motors are attached to thick filaments through an elastic element with asymmetric stiffness^37^ of 2 pN/nm in stretch and 0.4 pN/nm in contraction. At each time step, the algorithm updates the position of the i-th myosin, with respect to its anchor on the thick filament. The thin filament length is 1 µm long^38, 39^ and the thick filament is 1600 nm long with 200 nm of bare zone and with 49 myosin heads per half-thick filament, every 14.3 nm. Actually, 49 corresponds to the number of myosin molecules, each with two myosin motors, spaced by 42.9 nm and directly pointing to one actin filament, from the three thick filaments surrounding it (700/42.9) × 3^40^. We hypothesise that the two myosin motors cannot work at the same time, so the presence of two heads is modelled with a doubled probability of attaching to actin. The current position of the actin filament is the coupling factor for the myosin motors. The total active tension is given by the summation, for every attached myosin, of the product of its position times its stiffness. In the attached state, myosin motors are driven by a multi-stable potential energy with three minima^36^, corresponding to a pre-power stroke and a two-step power stroke. An energetic bias toward the next post-power stroke state of 10 k_B_T is imposed (k_B_ is the Boltzmann constant and T the absolute temperature). Distances between minima are assumed to be constant at 4.5 nm. Myosin motors can detach from any strongly-attached state. A mechanical detachment is imposed when myosin motors are stretched or compressed more than 20 nm.

A half-sarcomere is composed of 480 filaments. TT-units are placed every 36 nm on the thin filaments and can be inactive, partially active or active. The forward rates between these states are linearly dependent on [Ca^2+^], while the backward rates are constant. Thin and thick filaments are assumed to be rigid. An area of 1000 nm^2^ is assumed around each thin filament, leading to a tension of 1 kPa for each pN sustained by the thin filament itself. Then, for geometric reasons, the forces on the thick filament are twice those on the thin filament. Boundary and initial conditions in numerical simulations were chosen to be as similar as possible to the experimental protocols.

Attachment and detachment rates were chosen to fit the tension values obtained experimentally^10^ on rat trableculae at 27 °C. With these values, the maximum tension generated at high calcium levels is 120 kPa on the trabecula cross-sectional area. Isotonic contraction for the force–velocity curve was imposed during the simulated twitch, when the isometric contraction reached 95% of the maximum tension reached during the twitch, as in the experimental protocol. Model parameters are shown in Supplementary Table S1.

### Ventricle model

The beating heart simulation is performed with a rotationally symmetric left ventricle model, as shown in Supplementary Fig. S1 (see also Supplementary Information). The ventricular wall is simultaneously stimulated by the same time profile of calcium ion concentrations as in the single twitch simulation. The fiber orientation is twisted from 90 to −60 degrees against the circumferential direction, from the internal wall to the outer wall along the transmural direction. The parameters of the potentials which determine the passive stresses are selected to reproduce the SL–passive stress relationship reported previously for the mouse LV wall^7^. The diastolic pressure–volume relationship (shown in the inset of Supplementary Fig. S1) is reproduced. These results fit well with the experimental data^41^.

## Electronic supplementary material

Supplementary Informations
