# Supplementary material for: Titin-mediated thick filament activation, through a mechanosensing mechanism, introduces sarcomere-length dependencies in mathematical models of rat trabecula and whole ventricle
Source: Sci Rep. 2017 Jul 17;7:5546. doi: 10.1038/s41598-017-05999-2 (PMC5514028; doi:10.1038/s41598-017-05999-2)
Supplement: Supplementary file 1 — Supplementary Informations [file 41598_2017_5999_MOESM1_ESM.pdf]

Supplementary Information for:
Titin-mediated thick filament activation, through a mechanosensing mechanism, introduces sarcomere-length dependencies in mathematical models of rat trabecula and whole ventricle

Authors:
Lorenzo Marcucci, Takumi Washio, Toshio Yanagida

Ventricular model

The beating heart simulation is performed with a rotationally symmetric left ventricle model, as shown in Fig. S1. The ventricular wall is simultaneously stimulated by the same time profile of calcium ion concentration as the single twitch simulation. The momentum equation of the heart wall is given as follows.

$$\int_{\Omega_0} \delta\mathbf{u} \cdot \rho\ddot{\mathbf{u}} d\Omega + \int_{\Omega_0} \frac{\partial \delta\mathbf{u}}{\partial \mathbf{X}} : \mathbf{\Pi}\, d\Omega = \int_{\Gamma_t} P_{\mathrm{Cav}} \delta\mathbf{u} \cdot \mathbf{n} d\Gamma$$

Here, Ω_0 is the ventricle wall in the unloaded condition, $\mathbf{u} = \mathbf{u}(\mathbf{X}, t)$ is the displacement of the material point $\mathbf{X} \in \Omega_0$ at time t, and ρ is the density of the heart muscle ($\rho = 1.366 \mathrm{Kg/m^3}$). Γ_t is the blood–wall interface at time t and $\mathbf{n}$ is the outward normal vector on Γ_t. P_{Cav} is the intracavity pressure, which is determined by combining the conservation equation of the blood volume:

$$\frac{dV_{\mathrm{Cav}}}{dt} = \int_{\Gamma_t} \dot{\mathbf{u}} \cdot \mathbf{n} d\Gamma = F_{in} - F_{out},$$

where V_{Cav} is the intracavity volume, F_{in} is the inflow from the preload and the F_{out} is the outflow to the afterload. These flows are determined from the relationship between the intracavity pressure P_{Cav} and the pressure behind the valves, represented by the rectifiers in Fig. S1.

$\mathbf{\Pi}$ is the first Piola-Kirchhoff stress tensor, composed of active and passive stresses:

$$\mathbf{\Pi} = \mathbf{\Pi}_{act} + \mathbf{\Pi}_{pas}.$$

$\mathbf{\Pi}_{act}$ is derived from the active tension T with

$$\mathbf{\Pi}_{act} = \frac{T}{\lambda} \mathbf{f} \otimes \mathbf{f} \cdot \mathbf{F}^T,$$

where $\mathbf{F} = \mathbf{I} + \partial\mathbf{u} / \partial\mathbf{X}$ is the deformation gradient tensor and $\lambda = \|\mathbf{Ff}\|$ is the stretch along the myofibril fiber direction[1]. The fiber orientation is twisted from 90 to -60 degrees in the circumferential direction, from the internal wall to the outer wall, along the transmural direction. The passive stress is determined by the potential:

$$W_{pas} = W_{Mooney} + \kappa(\det(\mathbf{F}) - 1)^2 + W_{titin}(\lambda).$$

The first term represents the homogeneous potential of the Mooney-Rivlin body:

$$W_{Mooney} = c_1(\tilde{I}_1 - 3) + c_2(\tilde{I}_1 - 3)^2 + c_3(\tilde{I}_1 - 3)^2(\tilde{I}_2 - 3).$$

Here, $\widetilde{I}_1 = \det(\mathbf{C})^{-1/3}\,\mathrm{Tr}(\mathbf{C})$, $\widetilde{I}_2 = \det(\mathbf{C})^{-2/3}\left(\mathrm{Tr}(\mathbf{C})^2 - \mathrm{Tr}(\mathbf{C}^2)\right)$ represents reduced invariant determined by the right Cauchy-Green deformation tensor $\mathbf{C} = \mathbf{F}^T\mathbf{F}$ (c_1=40Pa, c_2=50Pa, c_3=1Pa). The second term is the potential for volumetric deformation ($\kappa = 10^6\,\mathrm{Pa}$). The third term represents the potential given by titin. This potential is a function of the stretch λ along the fiber direction. The parameters of this potential are determined as they reproduce the SL-passive stress relationship shown for the mouse LV wall by Granzier and collaborators[2]. In fact, with these passive stresses, the diastolic pressure–volume relationship, shown in the inset of Fig. S1, was reproduced. This result agreed well with the experimental data[3].

Hill's curve analysis

Starting with this form of Hill's equation[4]

$$y = \frac{T}{T_0} = \frac{[Ca^{2+}]^{nH}}{{pCa_{50}}^{nH} + [Ca^{2+}]^{nH}}$$

We can compute at which $[Ca^{2+}]_\alpha$ (or its logarithm) there would be a value of α between 0 and 1 as

$$\log([Ca^{2+}]_\alpha) = \frac{1}{nH} * [\log(\alpha) - \log(1-\alpha)] + \log(pCa_{50})$$

Given the symmetry of the equation, we can estimate the amplitude of the values of log(x) around k, at which the Hill's function is between α and (1-α). This indicates the slope of the curve.

$$[\log([Ca^{2+}]_\alpha) - \log(pCa_{50})]|_\alpha - [\log([Ca^{2+}]_\alpha) - \log(pCa_{50})]|_{1-\alpha} =$$

$$2/nH[\log(\alpha) - \log(1-\alpha)]$$

It is inversely proportional to nH. In our current model, the SL effect is irrelevant at high [Ca2+] because the high active tension, alone, is sufficient to maximally activate the myosin motors. At low [Ca2+], higher SLs lead to lower $\log([Ca^{2+}]_\alpha)$. Consequently, the fitted nH values decrease with SL.

Numerical simulation of state transitions

Numerical simulations of the state transitions of myosin motors are performed using a built-in pseudorandom number generator in FORTRAN90 (*rand*) and confronting it with the rate constant of the transition, multiplied by the time step of the simulation (Δt=1μs). Modifying the random seed slightly affects the simulation results, as shown in Figure S2 (twitches simulation at SL=2.2 μm with three different random seeds), with modifications well within

the semi-qualitative approach used in the paper and without modifying the general behavior simulated by the model. We then used the same number to initialize the pseudorandom number generator (random seed) for all simulation presented in this report.

1. Washio, T. *et al.* Ventricular fiber optimization utilizing the branching structure: Ventricular fiber optimization utilizing the branching structure. *Int. J. Numer. Methods Biomed. Eng.* **32,** e02753 (2016).
2. Granzier, H. L. & Labeit, S. The Giant Protein Titin. *Circ. Res.* **94,** 284–295 (2004).
3. Niederer, S. A. & Smith, N. P. The Role of the Frank–Starling Law in the Transduction of Cellular Work to Whole Organ Pump Function: A Computational Modeling Analysis. *PLoS Comput. Biol.* **5,** e1000371 (2009).
4. Walker, J. S., Li, X. & Buttrick, P. M. Analysing force–pCa curves. *J. Muscle Res. Cell Motil.* **31,** 59–69 (2010).

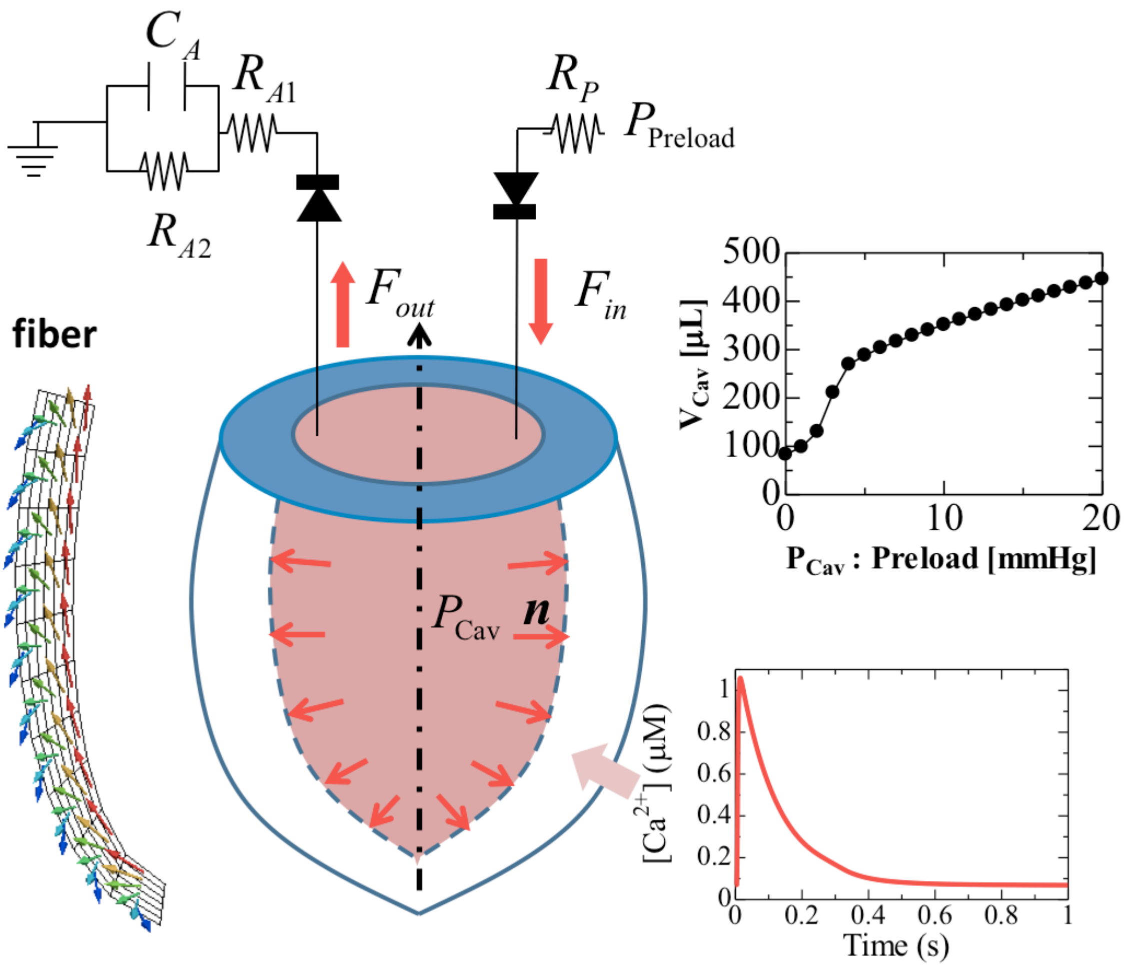

Figure S1: Ventricle model. Schematic representation of the rotational symmetric ventricle model and the circuits for inflow and outflow. The insets show the fiber directions (left), the volume–pressure relationships for the passive properties (upper right) and the $[Ca^{2+}]$ transients imposed in both the single fiber and the ventricle models (lower right).

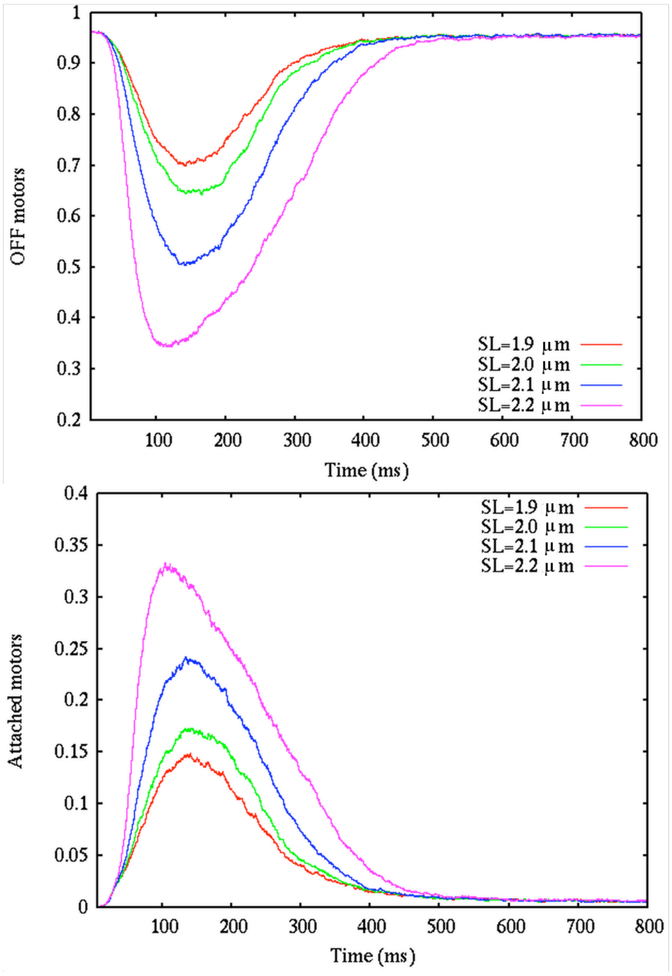

Figure S2: Relative number of OFF and attached motors during twitches at different SLs. In the model, passive tension at different SLs is always below the threshold tension to activate myosin motors above constitutively ON motors. Passive tension becomes important when thin filament activation increases the active tension. Increasing the SL from 1.9 to 2.2 µm, increases the passive tension of 0.075 T_0. This tension increases both the active (ON) motors and the force-generating (attached) motors, over two-fold.

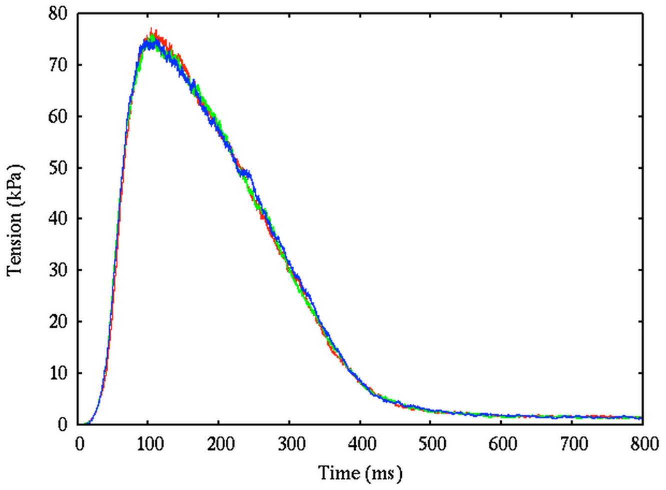

Figure S3: Tension vs. time at different random seeds. Simulation of the tension generated during a twitch at SL=2.2 μm, under three different values of the random seed. Numerical simulations for the state transitions of myosin motors were performed using a built-in pseudorandom number generator in FORTRAN90 (*rand*) and confronting it with the rate constant for each transition, multiplied by the time step of the simulation (Δt=1 μs). Modifying the random seed slightly affected the simulation results, with modifications well within the semi-qualitative approach used in the paper and without modifying the general behavior described by the model. All figures in the paper were generated using the same value to initialize the pseudorandom number generator (random seed).

Table S1: Single fiber model parameters

Parameter	Value	Meaning
N_{fil}	480	Number of filaments
N_{XB}	49	Myosin motors per filament
k	2-0.4 pN/nm	Myosin stiffness (stretched-compressed)
LA	1000 nm	Length of thin filament
LM	1600 nm	Length of thick filament
LB	200 nm	Length of bare zone in thick filament
k_{min}	10.2 s^{-1}	Minimum $k_{OFF\text{-}ON}$
k_{max}	442 s^{-1}	Maximum $k_{OFF\text{-}ON}$
T_{min}	17 kPa	Beginning tension for the linear part of $k_{OFF\text{-}ON}$
T_{max}	120 kPa	Ending tension for the linear part of $k_{OFF\text{-}ON}$
$k_{OFF\text{-}ON}$	Equation S1	OFF to ON rate constant
$k_{ON\text{-}OFF}$	262.4 s^{-1}	ON to OFF rate constant
$k_{ON\text{-}S}$	Equation S2	Attachment rate
$k_{S\text{-}ON}$	Equation S3	Detachment rate
α_-	421 s^{-1} nm^{-1}	Linear dependence on detachment
α_+	1022 s^{-1} nm^{-1}	Linear dependence on attachment
d_{min}	4.5 nm	Power stroke (PS) steps
a_{ATP}	10 K_BT	Energy drop, between prePS, PS1 and PS2
H	5 K_BT	Energy barrier, between prePS, PS1 and PS2
k^+_{Ca}	150 s^{-1} μM^{-1}	Attachment Ca^{2+} rate
k^-_{Ca}	48 s^{-1}	Detachment Ca^{2+} rate
μ_{Ca}	55	Reduction term in non-activated actin

$$k_{OFF\text{-}ON} = \begin{cases} k_{min} & T_{tot} < T_{min} \\ \frac{k_{max}\text{-}k_{min}}{T_{max}\text{-}T_{\min}}(T_{tot}\text{-}T_{min}) & T_{min} < T_{tot} < T_{max} \\ k_{max} & T_{tot} > T_{max} \end{cases} \quad \text{(S1)}$$

$$k_{ON\text{-}S}(x) = \begin{cases} \alpha_+ x & 0 < x < 10\ nm \\ 0 & elsewhere \end{cases} \quad \text{(S2)}$$

$$k_{S\text{-}ON}(x) = \begin{cases} \alpha_- x & x > 0 \\ 490\ s^{-1} & x < 0 \end{cases} \quad \text{(S3)}$$
